# Supplementary material for: Modeling the SARS-CoV-2 epidemic and the efficacy of different vaccines across different network structures
Source: PLoS One. 2025 Jun 5;20(6):e0325129. doi: 10.1371/journal.pone.0325129 (PMC12140218; doi:10.1371/journal.pone.0325129)
Supplement: Attachment — Submitted filename: Response to Reviewers.docx [file pone.0325129.s001.docx]

April 21, 2025

PLoS ONE (PONE-D-25-01005)

Dear David Mori˜na Soler and/or PLoS Editor(s):

We have submitted for your consideration the revised version of our paper titled “Modeling the SARS-CoV-2

Epidemic and the Efficacy of Different Vaccines Across Different Network Structures.” The paper includes

changes suggested by the editor and two reviewers. None of the suggestions is contested. Below are comments

of how we address each suggestion:

Comments by the Editor:

1. We have used latexdiff to generate a new manuscript with track changes.

2. We have removed the acknowledgement to the funding source. The only funding source is: “SUNY

Geneseo, Office of Sponsored Research.”

3. The code for the model (30 Kb) is on Dryad (https://doi.org/10.5061/dryad.98sf7m0vh) and remains

available on GitHub (https://github.com/GreggHartvigsen/Modeling-COVID-Vaccine-Efficacy/). Both

reviewers report that the data (code) have been made available.

4. The abstract is in the manuscript when we click “View Submission”. We have double-checked that the

abstract is the same in both the manuscript and the submission form.

5. We have reviewed the bibliography and fixed a missing reference citation.

6. We have standardized all parameters and state variables to be italicized (some were not).

7. We changed the amusing statement “With time vaccinated, individuals become less susceptible....” to

“With time, vaccinated individuals become less susceptible....”

8. Further, minor clarifications have been made along the lines of reviewer suggestions.

Comments by the Reviewer #1:

1. The reviewer asks for clarification on network structure. We have clarified the meaning of the rewiring

parameter P in the Methods section.

2. The reviewer expresses confusion as to why these particular network structures were used (e.g., P =

0.004). To help clarify this for the reader, we refer them to figure #2 which shows why these values

were chosen (highlighted as red bars). We clarify this in the manuscript.

3. We have clarified what is meant by “random vaccination” and the strategy that targets high degree

individuals.

4. We have extended our discussion of why the second vaccination dose did not have much of an effect,

in both the results and discussion sections.

Comments by the Reviewer #2:

1. We have added a discussion of the R0 values found in different countries to the Introduction and that

we decided to use relatively conservative values to test.

2. We have addressed the reason we set b = 1.75 in equation 2, which simply allows the second vaccine

does to continue the efficacy of the first dose.

3. In figure #6’s caption we changed “total number infected” to “total number of infectious individuals,”

an important distinction.

4. We added to the Introduction a mentioning of examples of small-world networks that exhibit similar

characteristics to the work in this manuscript. In addition, we added a discussion of estimating network

structure in real networks.

5. The second Acknowledgments section has been removed.

We thank you this opportunity to revise this manuscript.

Sincerely

Gregg Hartvigsen and Yannis Dimitroff

contact: hartvig@geneseo.edu
